# Supplementary material for: Exploring Optimal Biomarker Sources: A Comparative Analysis of Exosomes and Whole Plasma in Fasting and Non-Fasting Conditions for Liquid Biopsy Applications
Source: Int J Mol Sci. 2023 Dec 27;25(1):371. doi: 10.3390/ijms25010371 (PMC10779159; doi:10.3390/ijms25010371)

Supplementary Information

Exploring Optimal Biomarker Sources: A Comparative Analysis of Exosomes and Whole Plasma in Fasting and Non-Fasting Conditions for Liquid Biopsy Applications

Supplementary Table S1. Number of metabolite species detected by LC-MS/MS. We detected total of 513 metabolites from whole plasma samples and 261 from exosomes.

Type of metabolites	Plasma	Exosomes
Amino acids	30	13
Bile acids	35	3
Fatty acids	39	25
TG DG	80	61
Lipids	329	159

Supplementary Table S2. Metabolites species significantly affected by fasting/non-fasting status found by the volcano plot analysis.

	FC	log2(FC)	P value	Log10(p)	Type of metabolites
Beta_Alanine	2.0786	1.0556	0.0027003	2.5686	Amino acid
NorCA	3.079	1.6225	0.00096121	3.0172	Bile acid
alloLCA	0.53841	−0.89323	0.0044331	2.3533	Bile acid
GCDCA	3.018	1.5936	0.017655	1.7531	Bile acid
DCA	0.60349	−0.7286	0.024179	1.6166	Bile acid
GDCA	2.6626	1.4128	0.045147	1.3454	Bile acid
C8:0	1.8261	0.86876	0.0046764	2.3301	Fatty acid
C6:0	1.6173	0.69357	0.013595	1.8666	Fatty acid
C20:4(cis_5,8,11,14)	0.63922	−0.64561	0.029967	1.5234	Fatty acid
C22:4(cis_7,10,13,16)	0.60597	−0.72268	0.033014	1.4813	Fatty acid
C20:5(cis_5,8,11,14,17)	0.66825	−0.58154	0.033866	1.4702	Fatty acid
C22:6(cis_4,7,10,13,16,19)	0.51495	−0.95749	0.046803	1.3297	Fatty acid
C10:1	0.61041	−0.71214	0.0024672	2.6078	Lipids
LPE(18:3)	1.8587	0.89431	0.0032373	2.4898	Lipids
C24:5 CE	0.65995	−0.59958	0.0037706	2.4236	Lipids
PE(36:3)	2.22	1.1506	0.0059099	2.2284	Lipids
C8	0.51865	−0.94716	0.0059176	2.2279	Lipids
PE(34:0)	2.3969	1.2612	0.0065935	2.1809	Lipids
PE(36:1)	2.1074	1.0754	0.0067634	2.1698	Lipids
C14:2	0.57306	−0.80323	0.0074514	2.1278	Lipids
C10	0.48996	−1.0293	0.0078469	2.1053	Lipids
PE(34:1)	1.5045	0.58925	0.0084805	2.0716	Lipids
PE(36:5)	1.4196	0.50551	0.0085363	2.0687	Lipids
C12:1	0.62324	−0.68215	0.0093646	2.0285	Lipids
PE(34:2)	1.6736	0.74291	0.0096563	2.0152	Lipids
LPE(18:1)	1.5282	0.61179	0.0097845	2.0095	Lipids
C12	0.55351	−0.85332	0.010203	1.9913	Lipids
LPE(22:5)	1.4508	0.53685	0.010689	1.9711	Lipids
LPE(18:2)	1.649	0.72156	0.011611	1.9351	Lipids
C16:2	0.60373	−0.72803	0.012639	1.8983	Lipids
PE(36:2)	1.9388	0.95518	0.013554	1.8679	Lipids
PE(34:3)	1.7351	0.79504	0.013775	1.8609	Lipids
C14:1	0.57287	−0.80371	0.014334	1.8436	Lipids
PE(40:8)	1.8536	0.89034	0.019088	1.7192	Lipids
PE(32:2)	1.4159	0.50174	0.022035	1.6569	Lipids
PS(44:2)	1.5785	0.65852	0.024037	1.6191	Lipids
C18:1-OH	0.60669	−0.72097	0.027108	1.5669	Lipids
C14:1-OH	0.66287	−0.59319	0.028463	1.5457	Lipids
PE(42:8)	1.6385	0.71234	0.028693	1.5422	Lipids
PE(32:0)	1.7153	0.77843	0.037441	1.4267	Lipids
Cer(d18:0/16:0)	1.665	0.73552	0.040398	1.3936	Lipids
Cer(d18:0/24:0)	1.433	0.519	0.041156	1.3856	Lipids
PS(44:3)	1.7628	0.81788	0.041412	1.3829	Lipids
C7-DC	0.65193	−0.61721	0.043846	1.3581	Lipids
C16:1-OH	0.70403	−0.50629	0.04466	1.3501	Lipids

Supplementary Table S3. Percentage of unstable metabolites species affected by fasting/ non-fasting status detected in whole plasma. Fold-change within 1.4 is stable samples.

Type of metabolites	Number of Species	Stable Species	Unstable Species	% of Unstable
**TG/DG**	80	9	71	**88.75**
**Amino Acids**	30	28	2	**6.67**
**Bile Acids**	35	18	17	**48.57**
**Fatty Acids**	39	20	19	**48.72**
**Lipids**	329	292	37	**11.25**

Supplementary Table S4. Information of 4 Volunteers for blood collection.

Volunteer	Sex	Age	Race
A	Male	52	Asian
B	Male	38	Caucasian
C	Female	52	Asian
D	Male	30	Asian

Supplementary Figure S1. Volcano plots display the metabolite species of exosomes with significant changes after food consumption. Red dots: upregulation, Blue dots: downregulation, Gray: Non-significant changes. A) TG (Triacylglycerol)-DG (Diacylglycerol), B) Lipids.

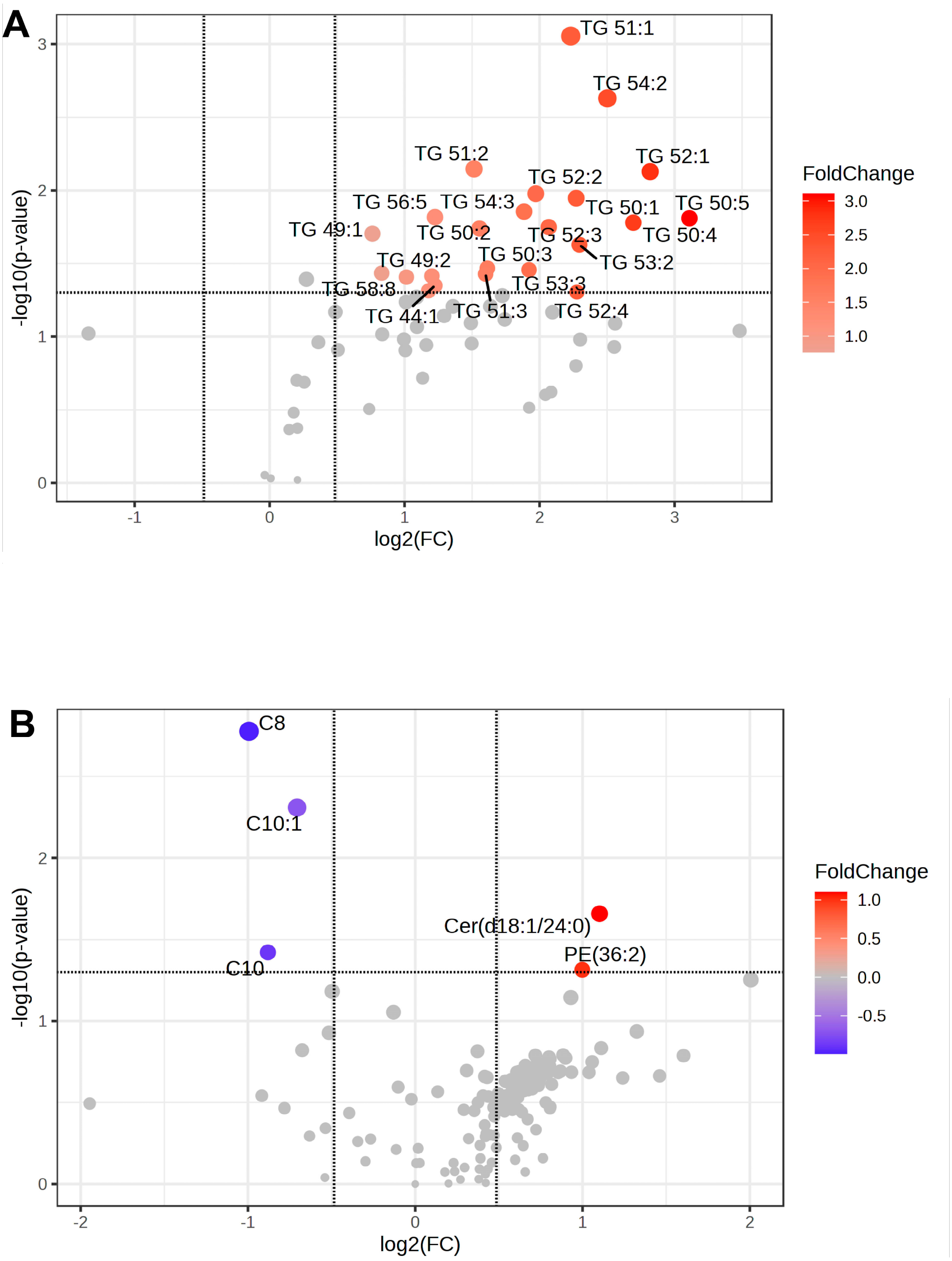

Supplementary Figure S2. Images of exosomes (EVs) extracted with the Capturem™ Extracellular Vesicle Isolation Kit (Cat. No. 635741, Takara Bio USA, Inc.) from our samples that were taken with a TEM (Transmission Electron Microscope) utilizing negative staining. 4μL of the sample was applied to the surface of a carbon and Formvar-coated grid for 45 seconds. Excess fluid was wicked away with a filter paper point. 10μL of distilled water was then applied, with the excess water being removed. 4μL (e.g., 2% uranyl acetate) was applied for 45 seconds. Excess fluid was wicked away again. 10μL of distilled water was applied for a second time. As the objective was to leave the same height of stain as the height of particles in the sample, excess fluid was carefully absorbed using a filter paper point. The goal was to keep only a very thin film of fluid on the grid, which would then be left to air dry.

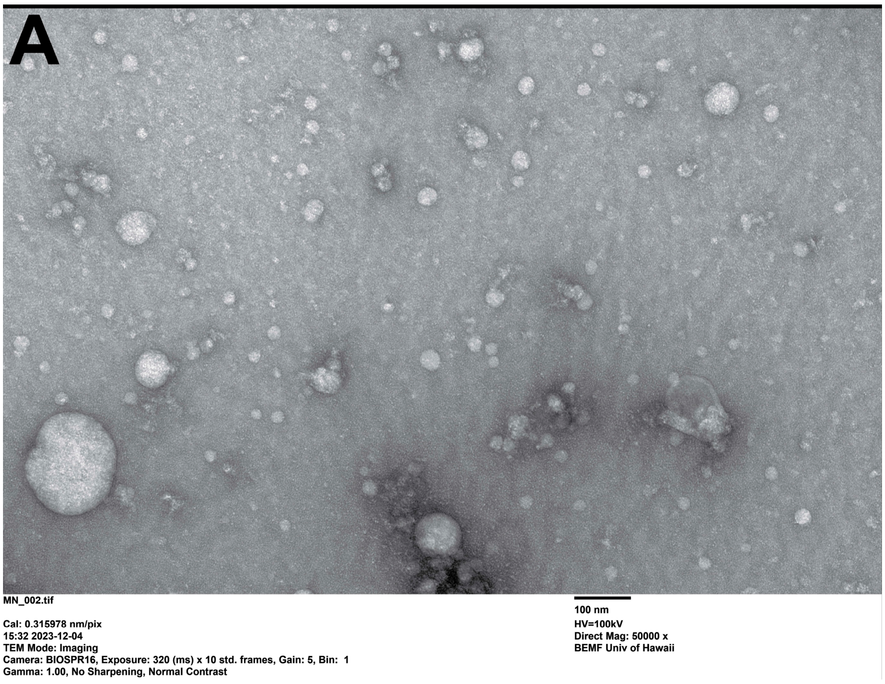

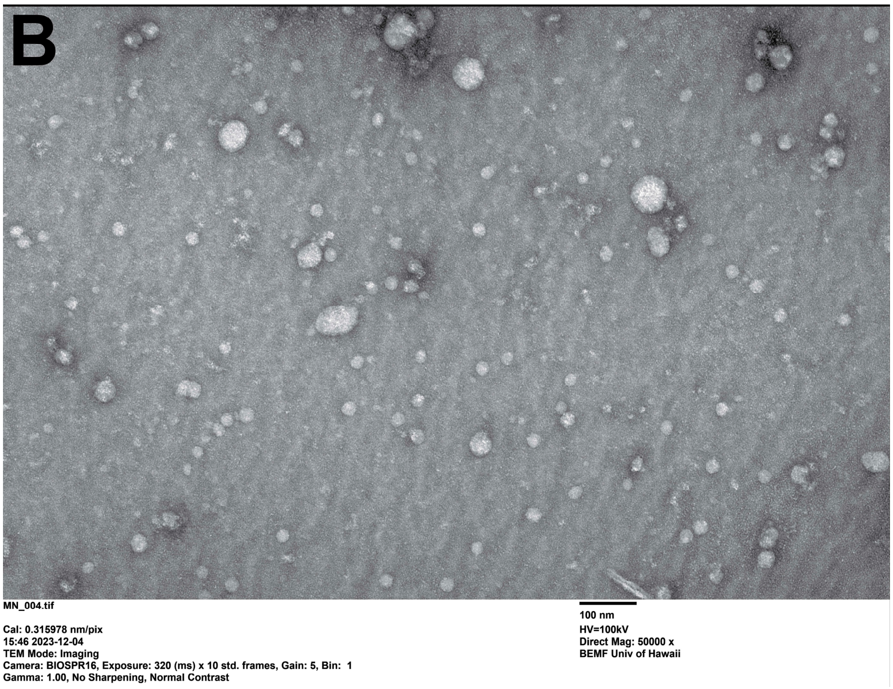

Supplement: Supplementary file 1 [file ijms-25-00371-s001.zip › ijms-2778616-supplementary.pdf]
